# Phosphorylation-facilitated sumoylation of MEF2C negatively regulates its transcriptional activity

**DOI:** 10.1186/1471-2091-7-5

**Published:** 2006-02-14

**Authors:** Jungseog Kang, Christian B Gocke, Hongtao Yu

**Affiliations:** 1 Department of Pharmacology, The University of Texas Southwestern Medical Center, 6001, Forest Park Road, Dallas, TX 75390-9041, USA

## Abstract

**Background:**

Sumoylation has emerged as an important posttranslational regulatory mechanism for transcription factors and cofactors. Sumoylation of many transcription factors represses their transcriptional activities. The myocyte enhancer factor 2 (MEF2) family of transcription factors plays an important role in regulating gene expression during myogenesis and has been recently shown to be sumoylated.

**Results:**

Consistent with earlier reports, we show that sumoylation of MEF2C at K391 inhibits its transcriptional activity. Sumoylation of MEF2C does not block its DNA-binding activity. A small C-terminal fragment of MEF2C containing K391, referred to as delta-N2-MEF2C, is efficiently sumoylated and, when targeted to DNA, represses transcription at neighbouring promoters. Because delta-N2-MEF2C lacks the binding site for class II histone deacetylases (HDACs), this result suggests that sumoylation of MEF2C may help to recruit transcriptional repressors other than these HDACs. Intriguingly, we show that phosphorylation of S396 in MEF2C, a residue in close proximity to the major sumoylation site (K391) and known to be phosphorylated *in vivo*, enhances sumoylation of delta- N2-MEF2C *in vitro*. The S396A mutation reduces sumoylation of MEF2C *in vivo *and enhances the transcription activity of MEF2C in reporter assays.

**Conclusion:**

We propose that phosphorylation of MEF2C at S396 facilitates its sumoylation at K391, which in turn recruits yet unidentified co-repressors to inhibit transcription. Our studies further suggest that sumoylation motifs containing a phosphorylated serine or an acidic residue at the +5 position might be more efficiently sumoylated.

## Background

Transcription factors and cofactors orchestrate complex yet precise programs of gene expression that are critical for cell proliferation and differentiation during development. The myocyte enhancer factor 2 (MEF2) family of transcription factors regulates diverse cellular processes in a wide range of cell types [1]. In muscle cells, the MEF2 family of proteins binds to the promoters of many muscle-specific genes and activates their transcription during muscle differentiation [2]. There are four MEF2 proteins, MEF2A, -B, -C, and -D, in vertebrates [1]. They contain a MADS box and an adjacent MEF2 motif at their N-termini that mediate DNA binding, homo- and hetero-dimerization, and cofactor binding [1,3,4]. They also contain a transcriptional activation domain at their C-termini [2]. Homozygous *mef2c *null mice are embryonic lethal due to defective cardiac myogenesis and morphogenesis [5,6] whereas *mef2a *null mice die suddenly shortly after birth due to defects in post-natal cardiomyocytes [7]. Therefore, among other functions, MEF2 proteins play essential and distinct roles during cardiac muscle development. In addition, mutations of human *MEF2A *have been implicated in the pathogenesis of a familial coronary artery disease [8].

The functions of MEF2 proteins are regulated by their direct physical interactions with a large collection of cofactors, including thyroid hormone receptor, MyoD, NFAT, GATA, p300, GRIP1, histone deacetylases (HDACs), and MITR [9-16]. The class II HDACs (HDAC4, -5, -7, and -9) contain a characteristic MEF2-interacting domain at their N-termini [3,4] and an HDAC catalytic domain at their C-termini. They bind to the MADS/MEF2 domain of MEF2 proteins and repress their transcriptional activity [13]. In addition to cofactor binding, MEF2 proteins are also regulated by multiple phosphorylation events [1]. Several kinases, including p38 MAP kinase, ERK5, and CDK5, can phosphorylate MEF2 proteins and regulate their transcriptional activity [17-19]. Phosphorylation of MEF2 by p38 occurs in the transcriptional activation domain of MEF2 and stimulates its transcriptional activity [19]. Recently, it has been shown that an alternative splicing event within the last coding exon of *MEF2C *leads to the formation of two MEF2C isoforms, only one of which contains a 32-residue domain called γ [20]. The MEF2C-(γ-) isoform has higher transcriptional activity than does MEF2C-(γ+), and a Gal4-γ-domain fusion protein represses the basal level transcription of a promoter with Gal4-binding sites [20]. Interestingly, Ser 396 within the γ-domain is phosphorylated and the S396A mutation diminishes the transcriptional repression activity of the γ-domain [20]. These results suggest that the γ-domain might recruit transcriptional repressors in a phosphorylation-dependent manner. Because a motif similar to the γ-domain is constitutively present in MEF2A, -B, and -D, phosphorylation within this motif might negatively regulate the transcriptional activities of other MEF2 proteins.

Small Ubiquitin-like Modifier (SUMO) is structurally related to ubiquitin and is covalently conjugated to lysine residues of target proteins through an amide bond [21,22]. There are at least three mammalian SUMO proteins, SUMO1, -2, and -3. SUMO2 and -3 share greater than 90% sequence identity while both are about 50% identical to SUMO1 [21,22]. Sumoylation is catalyzed by a set of enzymes, including E1-activating enzyme (AOS1/UBA2), E2-conjugating enzyme (UBC9), and E3 ligases [21,22]. Three types of SUMO E3 ligases, RanBP2, the PIAS proteins, and Pc2, have been identified [21,22]. They exhibit different subcellular localization patterns and might enhance sumoylation of specific subsets of SUMO substrates *in vivo *[23-26]. Like ubiquitination, sumoylation is a dynamic process and is actively reversed by SUMO-specific proteases, including SENP1, -2, -3 and -6 [21,22]. Unlike ubiquitination, sumoylation does not generally lead to degradation of target proteins [21,22]. Instead, sumoylation regulates the functions of substrate proteins in multiple ways, including controlling their subcellular localization, affecting their interactions with other proteins, or increasing their stability through antagonizing ubiquitination [21,22]. Many transcriptional factors and cofactors are sumoylated and, in most cases, sumoylation inhibits their transcriptional activity [21,22,27-35]. Sumoylation of Elk-1 and p300 enhances their binding to HDAC2 and -6, respectively, suggesting that sumoylation might repress transcription through recruitment of transcriptional repressors, such as HDACs [36,37]. However, it remains to be established whether HDAC recruitment is a general mechanism for transcriptional repression by sumoylation.

We have previously identified human MEF2 proteins as SUMO1 substrates using an *in vitro *expression cloning (IVEC) strategy [38]. In addition, MEF2C and MEF2D have very recently been shown to be modified by SUMO2 and SUMO3 [39]. Sumoylation of MEF2 is enhanced by class IIa HDACs and by SIRT1-mediated deacetylation of the lysine acceptor for SUMO [39,40]. Here we further confirm the sumoylation of MEF2C *in vivo *and identify K391 as the major sumoylation site of MEF2C. As compared to the wild-type MEF2C, the MEF2C-K391R mutant has higher transcriptional activity in reporter assays, and is more efficient in promoting the conversion of 10T1/2 cells into myocytes when co-expressed with MyoD. These findings suggest that sumoylation of MEF2C inhibits its transcriptional activity. Sumoylation of MEF2C does not appear to affect its binding to DNA, its interactions with HDACs, or its phosphorylation by p38. Interestingly, K391 is located in the γ-domain and in close proximity to S396, which can be phosphorylated by unknown kinase(s) [20]. When fused to Gal4, a C-terminal fragment of MEF2C containing the γ-domain is sufficient to repress transcription of a promoter that contained Gal4-binding sites. Mutations of either K391 or S396 abolish the transcriptional repression activity of the Gal4-MEF2C fusion protein. Moreover, phosphorylation of S396 enhances the sumoylation of the C-terminal domain of MEF2C *in vitro*. The S396A mutation reduces the degree of MEF2C sumoylation *in vivo*. We propose that phosphorylation of S396 facilitates the sumoylation of MEF2C, which in turn recruits yet unidentified transcriptional repressors to inhibit transcription.

## Results

### MEF2C is sumoylated at K391 in vivo

**Figure 1:**
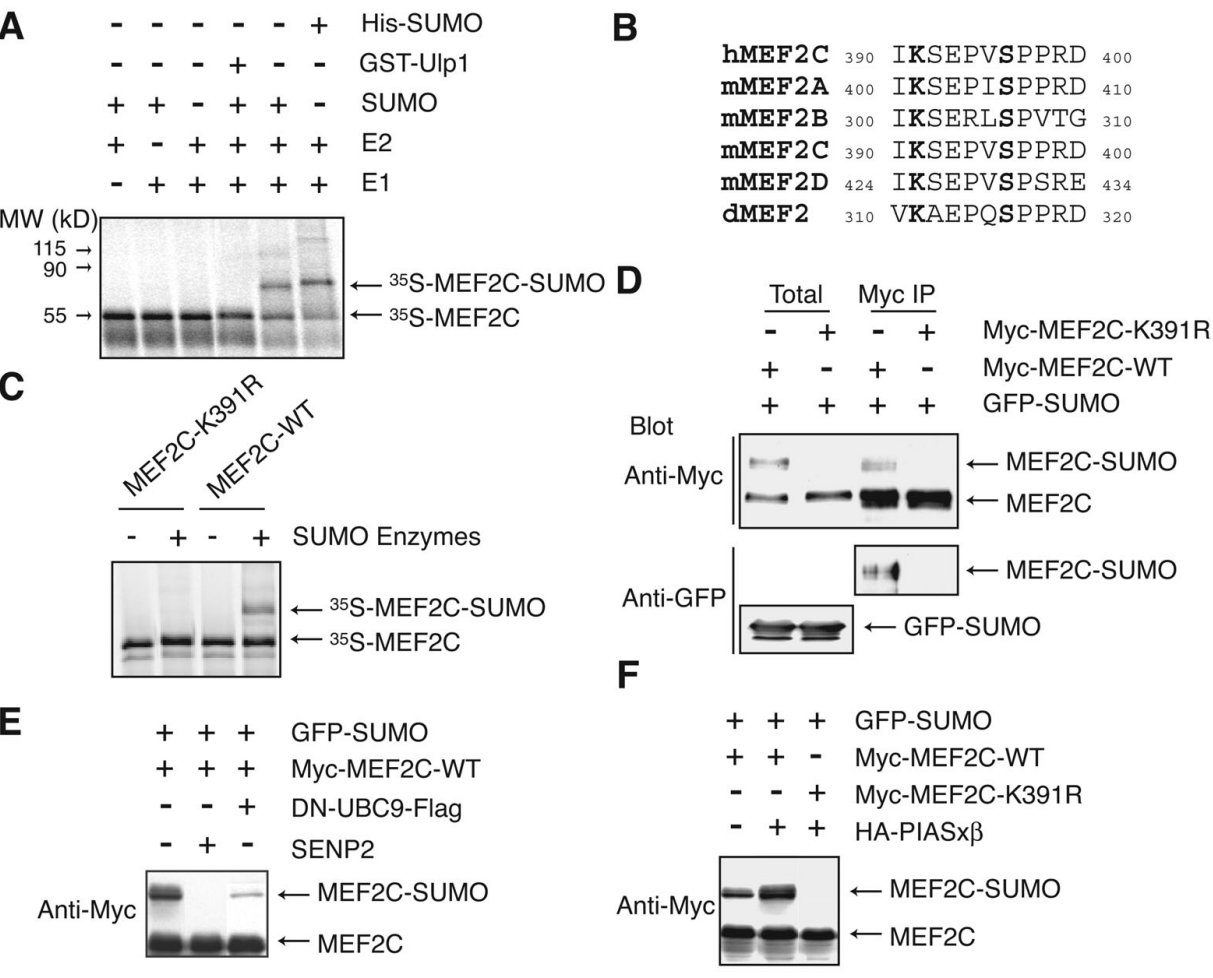
**MEF2 proteins are sumoylated**. **(A) **The ^35^S-labeled MEF2C protein obtained through in vitro transcription and translation was incubated with SUMO reaction mixtures and analyzed by SDS-PAGE followed by autoradiography. **(B) **Sequence alignment of the MEF2 family of proteins. The lysine residue of a sumoylation consensus motif and a serine residue that is phosphorylated are shown in bold. **(C) **The ^35^S-labeled wild-type (WT) and K391R mutant of MEF2C were incubated with SUMO reaction mixtures and analyzed by SDS-PAGE followed by autoradiography. **(D) **HeLa cells were transfected with the indicated plasmids. Myc-MEF2C was immunoprecipitated with anti-Myc. Cell lysates and Myc IP were resolved by SDS-PAGE and blotted with anti-Myc or anti-GFP. **(E) **HeLa cells were transfected with the indicated plasmids. Cell lysates were resolved by SDS-PAGE and blotted with anti-Myc. **(F) **HeLa cells were transfected with the indicated plasmids. Cell lysates were resolved by SDS-PAGE and blotted with anti-Myc.

We and Gregoire *et al*. had previously shown that the MEF2 family of transcription factors was efficiently sumoylated [38,39]. As shown in Figure 1A, MEF2C was modified in the presence of E1 (AOS1/UBA2), E2 (UBC9), and SUMO1. His_6_-SUMO1 resulted in a further shift in gel mobility as compared to untagged SUMO1 (Figure 1A). The yeast SUMO isopeptidase, Ulp1, efficiently reversed this modification (Figure 1A). MEF2C contains a sumoylation motif, ΦKXE (Φ, hydrophobic residues; X, any residues) [41,42] in the γ-domain of MEF2C [20] that is highly conserved among other members of the MEF2 family (Figure 1B). We mutated the lysine residue in this motif (K391) to arginine and observed the MEF2C-K391R is no longer sumoylated *in vitro *(Figure 1C). We then examined the sumoylation of MEF2C in HeLa and NIH3T3 cells (Figure 1D and data not shown). A significant fraction of Myc-MEF2C, but not Myc-MEF2C-K391R, was converted to a slow-migrating species when it was co-expressed with GFP-SUMO1 (Figure 1D, top panel). We then immunoprecipitated Myc-MEF2C and blotted the immunoprecipitates with anti-GFP. The slower migrating band contained GFP-SUMO1 (Figure 1D, middle panel). Furthermore, we observed that overexpression of SUMO isopeptidase, SENP2, or a dominant-negative mutant of UBC9 (DN-UBC9) greatly reduced the intensity of this slow-migrating band of Myc-MEF2C (Figure 1E). Co-expression of the PIAS family of E3 ligases, PIASxβ, enhanced the sumoylation of Myc-MEF2C-WT (Figure 1F), but not that of Myc-MEF2C-K391R. These data indicate that MEF2C is sumoylated *in vivo *and K391 is the major sumoylation site of MEF2C in living cells.

### Sumoylation of MEF2C reduces its transcriptional activity

**Figure 2:**
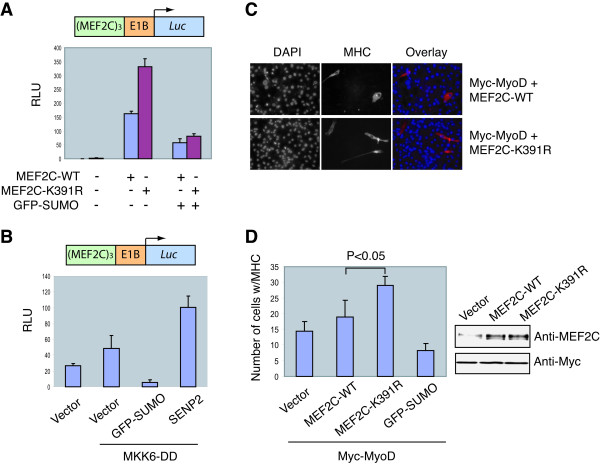
**Sumoylation-deficient mutant of MEF2C promotes myogenic conversion more efficiently**. **(A) **MEF2C-WT- or MEF2C-K391R-expressing plasmids were co-transfected with *MEF2*×3-luciferase reporter, pRL-*tk *reporter, and GFP-SUMO1 or empty vector plasmids into HeLa cells. Firefly luciferase activities were measured and normalized for transfection efficiency by using *Renilla *luciferase activities. **(B) ***MEF2*×3-luciferase reporter, pRL-*tk*reporter, and MKK6-DD plasmids were co-transfected with GFP-SUMO1, SENP2, or empty vector plasmids into C2C12 cells. The cells were cultured in differentiation medium for 2 days. Firefly luciferase activities were measured and normalized for transfection efficiency by using *Renilla *luciferase activities. **(C) **Vector, MEF2C-WT-, MEF2C-K391R-, or GFP-SUMO1-expressing constructs were co-transfected with Myc-MyoD into 10T1/2 cells. The cells were cultured in differentiation medium for 5 days, fixed, and stained with DAPI (blue) and an anti-myosin heavy chain (MHC) monoclonal antibody (red). **(D) **MHC-positive cells were scored by random selection of 20 optical fields of cells in **(C)**. The results of two independent experiments were averaged with the standard deviation indicated. P value was calculated using student t test. Cell lysates were resolved by SDS-PAGE and blotted with anti-MEF2C or Myc.

We next used a luciferase reporter assay to examine whether sumoylation of MEF2C regulates its transcriptional activity. The luciferase reporter construct contained three tandem copies of MEF2-binding sites at the promoter region. The transcriptional activity of MEF2C-K391R (the sumoylation-deficient mutant) was about two-fold higher than that of the wild-type MEF2C (Figure 2A), suggesting that sumoylation of MEF2C inhibits its transcription activity. Overexpression of GFP-SUMO1 downregulates the transcription activity of both MEF2C-WT and K391R (Figure 2A). Because overexpression of GFP-SUMO1 caused a global increase in the sumoylation of many cellular proteins (data not shown), inhibition of MEF2C-K391R by GFP-SUMO1 overexpression was most likely due to the enhanced sumoylation of other MEF2C regulatory proteins under these conditions. However, we cannot completely rule out the possibility that MEF2C is sumoylated at a second site, the sumoylation of which is below the detection limit of our assay.

We next examined whether sumoylation affected the activation of endogenous MEF2C by the p38MAPK pathway. To do so, we transfected a constitutively active mutant of MKK6 (an upstream activator of p38MAPK) into C2C12 cell lines, together with GFP-SUMO1 or SENP2. Overexpression of GFP-SUMO1 reduced the transcriptional activity of MEF2C whereas overexpression of SENP2 enhanced the activity of MEF2C in the presence of constitutively active MKK6 (Figure 2B). These results are consistent with the notion that sumoylation of MEF2C inhibits the transcription activity of the endogenous MEF2C stimulated by the p38MAPK pathway. However, it is entirely possible that alteration of the sumoylation levels of other MEF2C regulatory proteins is responsible for the observed effects of GFP-SUMO and SENP2.

Embryonic fibroblast cells can be converted into myoblasts upon overexpression of MyoD and MEF2 [13,43]. We tested whether MEF2C-K391R was more active than MEF2C-WT in collaborating with MyoD to promote the conversion of 10T1/2 cells into myoblasts. We transfected MyoD- and MEF2C-expressing plasmids into 10T1/2 cells and cultured these cells in low-serum media to induce the differentiation of the converted myoblasts into myotubes. On the fifth day after the induction of differentiation, the cells were fixed and stained with an antibody against myosin heavy chain (MHC), a well-established myogenic differentiation marker (Figure 2C and 2D). Co-expression of MEF2C-WT together with MyoD slightly increased the number of myotubes, as compared to the expression of MyoD alone (Figure 2D). Co-expression of MEF2C-K391R with MyoD increased the myoblast conversion rate by two-fold (P < 0.05) (Figure 2D). The expression levels of MEF2C-WT and MEF2C-K391R were similar (Figure 2D, left panel). These results suggest that sumoylation of MEF2C down-regulates its transcriptional activity during muscle differentiation.

### Sumoylation of MEF2C recruits transcriptional repressors other than Class II HDACs

We next sought to determine the mechanism by which sumoylation inhibits the transcriptional activity of MEF2C. We first tested whether sumoylation reduced the DNA-binding activity of MEF2C using the chromatin immunoprecipitation (ChIP) assay. Similar amounts of MEF2C-WT or MEF2C-K391R bound to the desmin promoter in a reporter plasmid [see Additional file 1], suggesting that sumoylation does not affect the DNA-binding activity of MEF2C. We next tested whether sumoylation of MEF2C increases its binding to HDACs, such as HDAC4 and HDAC5. MEF2C-WT and MEF2C-K391R bound equally well to HDAC4 or HDAC5 [see Additional file 1], suggesting that sumoylation of MEF2C does not recruit class II HDACs.

Since sumoylation inhibits the activation of the endogenous MEF2C by the p38MAPK pathway (Figure 2B), we thus tested whether sumoylation and p38 phosphorylation of MEF2C are antagonistic. However, we observed that sumoylation does not affect the binding affinity of MEF2C toward p38 or phosphorylation of MEF2C by p38 [see Additional file 1]. Likewise, phosphorylation of MEF2C by p38 does not appear to block sumoylation of MEF2C *in vivo *[see Additional file 1]. Thus, there is no significant interplay between sumoylation and p38 phosphorylation of MEF2C.

**Figure 3:**
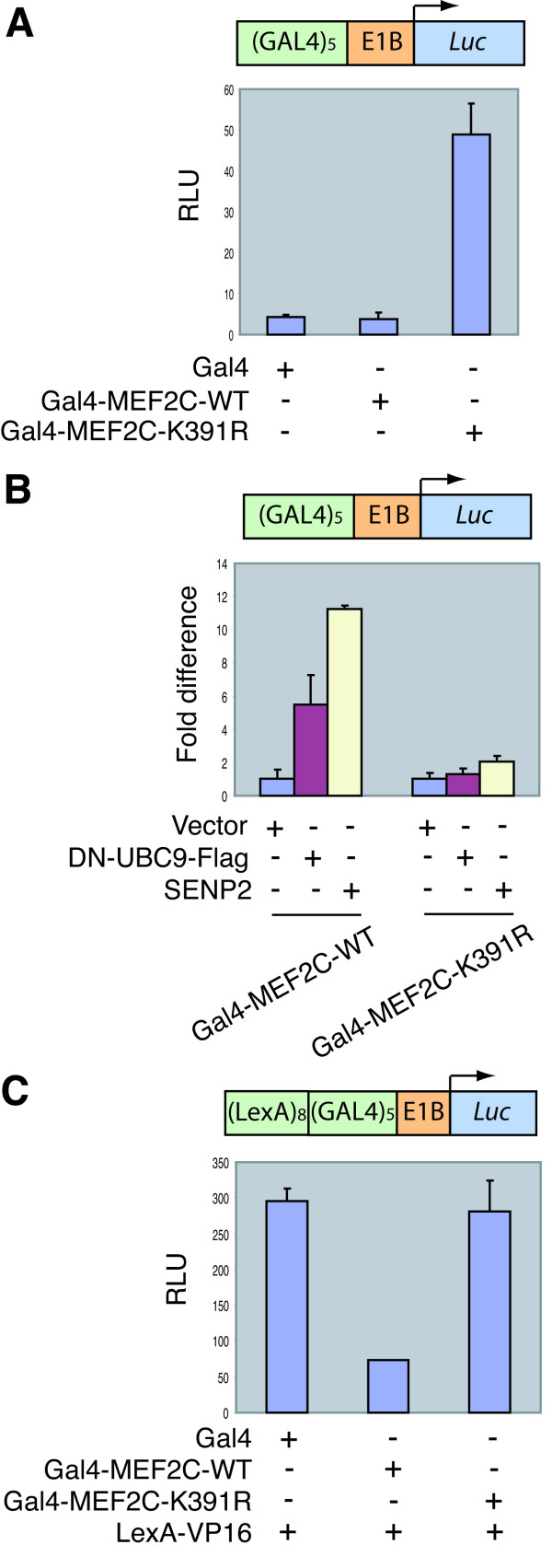
**Sumoylation of MEF2C inhibits its transcriptional activity**. **(A) **Gal4, Gal4-MEF2C-WT, or Gal4-MEF2C-K391R construct was co-transfected with *GAL4*×5-luciferase reporter and pRL-*tk *reporter into HeLa cells. Firefly luciferase activities were measured and normalized for transfection efficiency by using *Renilla *luciferase activities. **(B) **Gal4-MEF2C-WT or Gal4-MEF2C-K391R construct was co-transfected with *GAL4*×5-luciferase reporter, pRL-*tk *reporter, and SENP2, DN-UBC9-Flag, or vector construct into HeLa cells. Firefly luciferase activities were measured and normalized for transfection efficiency by using *Renilla *luciferase activities and then divided by the luciferase activities of Gal4-MEF2C-WT or Gal4-MEF2C-K391R, respectively, to show the fold differences. **(C) **Gal4, Gal4-MEF2C-WT, or Gal4-MEF2C-K391R construct was co-transfected with *LexA*×8-*GAL4*×5-luciferase reporter, pRL-*tk *reporter, and LexA-VP16 construct into HeLa cells. Firefly luciferase activities were measured and normalized for transfection efficiency by using *Renilla *luciferase activities.

To further study how sumoylation reduced the transcriptional activity of MEF2C, we measured the transcriptional activity of Gal4 fusion proteins of MEF2C and MEF2C-K391R, using luciferase reporter assays with a reporter construct that contained Gal4-binding sites. As compared to Gal4-MEF2C-WT, Gal4-MEF2C-K391R was much more active in stimulating transcription (Figure 3A). Overexpression of DN-UBC9 or SENP2 greatly increased the transcriptional activity of Gal4-MEF2C-WT, but not Gal4-MEF2C-K391R (Figure 3B). These data indicate that sumoylation also inhibits the transcriptional activity of MEF2C at a promoter that does not contain MEF2C-binding sites, consistent with the fact that sumoylation does not affect the DNA-binding activity of MEF2C.

We next performed luciferase reporter assays with a reporter construct that contained both Gal4- and LexA-binding sites. LexA-VP16 (a fusion protein of the LexA DNA-binding domain and the VP16 transactivation domain) dramatically stimulated the transcription of this reporter gene (Figure 3C). Co-expression of Gal4-MEF2C-WT, but not Gal4-MEF2C-K391R, greatly reduced the transcriptional activity of LexA-VP16 (Figure 3C). This suggests that Gal4-MEF2C might recruit transcriptional repressors to this artificial promoter in a manner that is dependent on its sumoylation.

**Figure 4:**
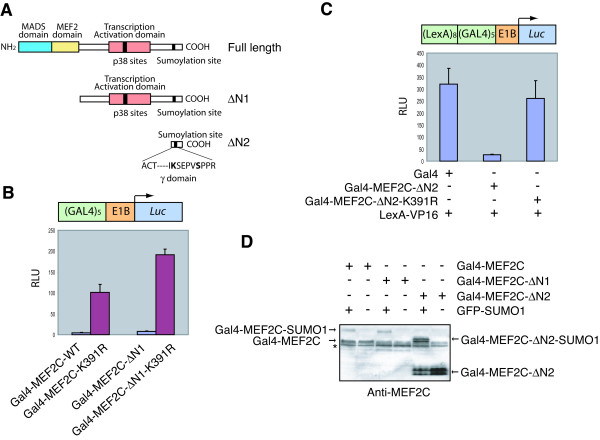
**A small C-terminal fragment of MEF2C is sufficient to repress transcription in a sumoylation-dependent manner**. **(A) **Schematic drawing of the functional domains and two C-terminal fragments of MEF2C. **(B) **Gal4-MEF2C-WT, Gal4-MEF2C-K391R, Gal4-MEF2C-ΔN1, or Gal4-MEF2C-ΔN1-K391R construct was co-transfected with *GAL4*×5-luciferase reporter and pRL-*tk *reporter constructs into HeLa cells. Firefly luciferase activities were measured and normalized for transfection efficiency by using *Renilla *luciferase activities. **(C) **Gal4, Gal4-MEF2C-ΔN2, or Gal4-MEF2C-ΔN2-K391R construct was cotransfected with *LexA*×8-*GAL4*×5-luciferase reporter, pRL-*tk *reporter, and LexA-VP16 constructs into HeLa cells. Firefly luciferase activities were measured and normalized for transfection efficiency by using *Renilla *luciferase activities. **(D) **HeLa cells were transfected with the indicated plasmids. The total cell lysates were resolved by SDS-PAGE and blotted with anti-MEF2C. The asterisk indicates the endogenous MEF2 proteins.

Since sumoylation of MEF2C does not appear to affect its binding to HDAC4 and HDAC5, recruitment of these HDACs is unlikely to be responsible for the transcriptional repression activity of Gal4-MEF2C. To further test this notion, we constructed Gal4-fusion proteins of two C-terminal MEF2C fragments, termed ΔN1 and ΔN2, and their corresponding K391R mutants, and tested their abilities to stimulate/repress transcription in reporter assays (Figure 4A). MEF2C-ΔN1 lacked the N-terminal MADS/MEF2 domain that is required for HDAC-binding whereas ΔN2 lacked both the MADS/MEF2 domain and the transactivation domain (Figure 4A). As shown in Figure 4B, Gal4-MEF2C-ΔN1-K391R was much more active in promoting transcription of the reporter gene that contained Gal4-binding sites at its promoter, suggesting that MEF2C-ΔN1 retained the ability to be regulated by SUMO. Furthermore, Gal4-MEF2C-ΔN2, but not Gal4-MEF2C-ΔN2-K391R repressed the activity of LexA-VP16 to stimulate transcription of the reporter gene whose promoter contained both Gal4- and LexA-binding sites (Figure 4C). Expectedly, both GAL4-MEF2C-ΔN1 and Gal4-MEF2C-ΔN2 were sumoylated in HeLa cells when co-expressed with GFP-SUMO1 (Figure 4D). Since MEF2C-ΔN1 and MEF2C-ΔN2 lack the HDAC-binding domain of MEF2C, these data suggest that sumoylation of MEF2C might mediate the recruitment of transcriptional repressors other than class II HDACs to repress the transcriptional activities of Gal4-MEF2C or LexA-VP16. In addition, MEF2C-ΔN2 lacks the p38 phosphorylation sites and yet retains the ability to be regulated by sumoylation. This is consistent with the notion that sumoylation does not repress the transcriptional activity of MEF2C through reducing p38 phosphorylation of MEF2C.

### Phosphorylation of S396 facilitates sumoylation of MEF2C

**Figure 5:**
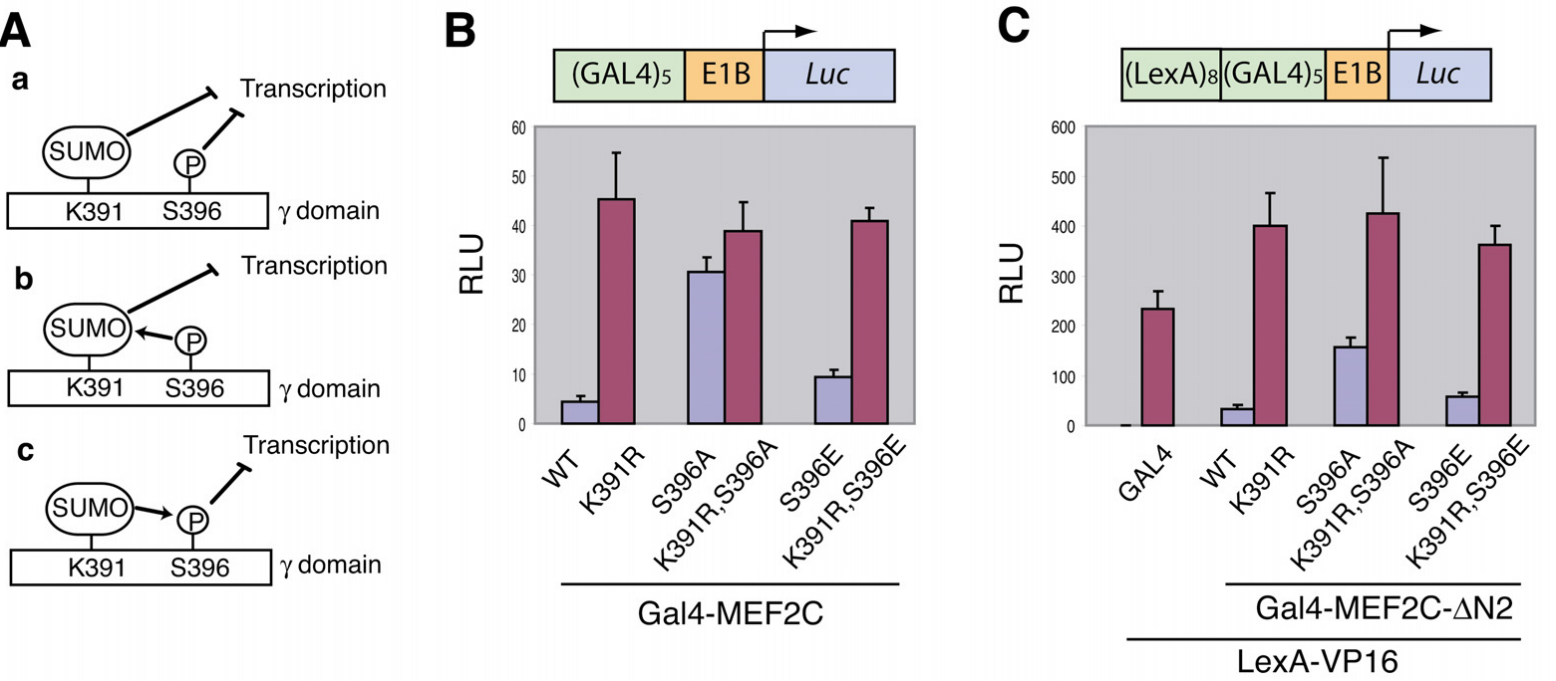
**Phosphorylation of MEF2C at S396 and sumoylation of MEF2C at K391 repress transcription through the same pathway**. **(A) **Three possible models with respect to the relationship between phosphorylation and sumoylation of MEF2C. **(B) ***GAL4*×5-luciferase reporter and pRL-*tk *reporter constructs were co-transfected with various Gal4-MEF2C constructs into HeLa cells. Firefly luciferase activities were measured and normalized for transfection efficiency by using *Renilla *luciferase activities. **(C) ***LexA*×8-*GAL4*×5-luciferase reporter, pRL-*tk *reporter, and LexA-VP16 constructs were cotransfected with various Gal4-MEF2C constructs into HeLa cells. Firefly luciferase activities were measured and normalized for transfection efficiency by using *Renilla *luciferase activities.

Recently, S396, a conserved serine residue in the C-terminal γ-domain of MEF2C, has been shown to be phosphorylated [20]. MEF2C-S396A or a MEF2C splicing variant that lacks the γ-domain is much more active in transcriptional reporter assays [20]. Moreover, γ-domain alone when fused to Gal4 can repress the basal transcriptional activity of Gal4, and this repression is diminished by the S396A mutation [20]. These findings indicate that phosphorylation of S396 inhibits the transcriptional activity of MEF2 proteins by recruiting transcriptional repressors. Interestingly, the major sumoylation site of MEF2C, K391, is located in the vicinity of S396 (Figure 1B and 5A).

We therefore tested whether phosphorylation of S396 affected the sumoylation of MEF2C. We envisioned three possible scenarios (**a**, **b**, and **c**) with respect to the relationship between phosphorylation of S396 and sumoylation at K391 (Figure 5A). In model **a**, phosphorylation and sumoylation are independent of each other. In this case, mutations of K391R and S396A are expected to have additive or synergistic effects in reporter assays. In models **b **and **c**, sumoylation of K391 is dependent on phosphorylation of S396, or vice versa. Gal4-MEF2C-K391R activated the transcription of a reporter gene that contained Gal4-binding sites much more efficiently than did Gal4-MEF2C-WT (Figure 5B). The Gal4-MEF2C-K391R,S396A double mutant did not exhibit higher transcriptional activity than did Gal4-MEF2C-K391R (Figure 5B). Similarly, both Gal4-MEF2C-ΔN2-K391R and Gal4-MEF2C-ΔN2-K391R,S396A completely lost the ability to repress the transcriptional activity of LexA-VP16 toward a reporter gene that contained both Gal4- and LexA-binding sites (Figure 5C). These data are consistent with the notion that phosphorylation of S396 and sumoylation of K391 act through the same pathway to repress transcription, and model **a **is unlikely to be correct. Furthermore, Gal4-MEF2C-S396A was less active in promoting transcription than Gal4-MEF2C-K391R (Figure 5B). Similarly, Gal4-MEF2C-ΔN2-S396A still retained partial activity in repressing the transcriptional activity of LexA-VP16 (Figure 5C). Although MEF2C-S396E (a phospho-mimicking mutant) was slightly more active than the MEF2C-WT for unknown reasons (see Discussion), it was much less active than the S396A mutant (Figure 5B and 5C). While we cannot rule out model **c**, our data are more consistent with model **b**, in that sumoylation of MEF2C is the key event in inhibiting its transcriptional activity and phosphorylation of S396 might facilitate the sumoylation of MEF2C at K391.

**Figure 6:**
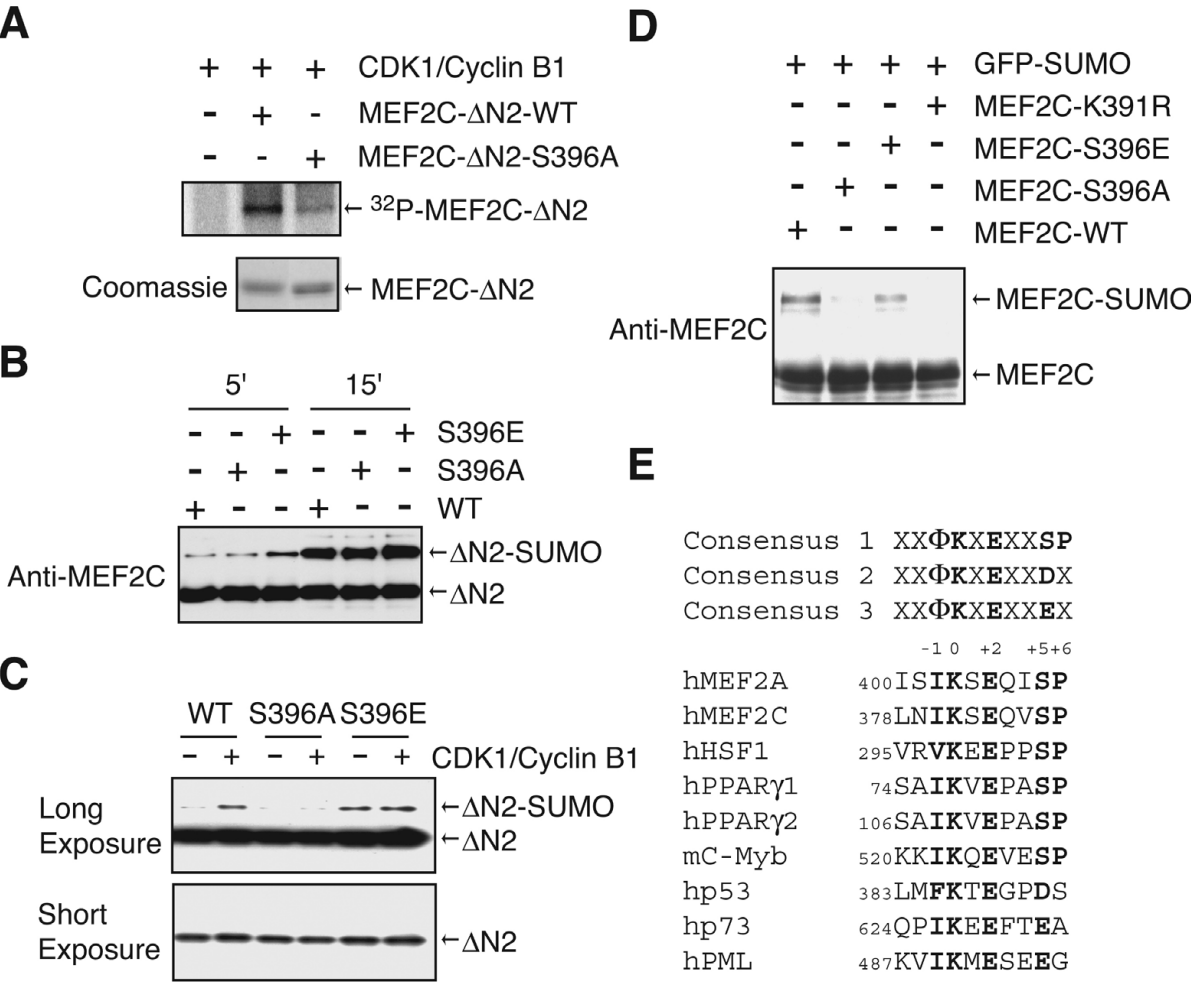
**MEF2C-S396A is less efficiently sumoylated *in vitro *and *in vivo***. **(A) **The recombinant GST-MEF2C-ΔN2-WT or GST-MEF2C-ΔN2-S396A proteins were subjected to CDK1 kinase assays and analyzed by SDS-PAGE followed by autoradiography. **(B) **0.75 μg of GST-MEF2C-ΔN2-WT, S396A, or S396E proteins were incubated with 1 μg of E1, 0.5 μg of E2, and 1.25 μg of His_6_-SUMO1 for the indicated time and stopped by SDS-PAGE sample buffer. The samples were resolved by SDS-PAGE and blotted with anti-MEF2C. **(C) **0.3 μg of GST-MEF2C-ΔN2-WT, S396A, or S396E proteins were subjected to CDK1 kinase assays and then incubated with 0.2 μg of E1, 0.1 μg of E2, and 0.3 μg of His_6_-SUMO1 for 15 min and stopped by SDS-PAGE sample buffer. The samples were resolved by SDS-PAGE and blotted with anti-MEF2C. **(D) **10T1/2 cells were transfected with the indicated plasmids. Cell lysates were resolved by SDS-PAGE and blotted with anti-MEF2C. **(E) **Sequence alignment of the sumoylation sites of several SUMO substrates. Conserved amino acids were shown in bold.

To directly test this hypothesis, we expressed and purified GST-MEF2C-ΔN2 and the corresponding S396A and S396E mutants from *E. coli *and examined their phosphorylation by Cdk1/Cyclin B1. CDK1/Cyclin B1 phosphorylated GST-MEF2C-ΔN2-WT to a greater extent than the MEF2C-ΔN2-S396A mutant (Figure 6A), suggesting that Cdk1/Cyclin B1 can phosphorylate MEF2C at S396. We then examined the sumoylation of recombinant GST-MEF2C-ΔN2-WT, S396A, and S396E proteins *in vitro*. As shown in Figure 6B, at high concentrations of SUMO reaction components and these substrate proteins, all three GST-MEF2C-ΔN2 proteins were efficiently sumoylated *in vitro*. The S396E mutant was slightly more efficiently sumoylated (Figure 6B). We next tested whether phosphorylation of MEF2C-ΔN2 by Cdk1 affected its sumoylation. Because sumoylation of the S396A mutant was already very efficiently sumoylated at high concentrations of SUMO enzymes, we performed the SUMO reactions with much lower concentrations of SUMO reaction components and substrates. Under these conditions, addition of Cdk1/Cyclin B1 enhanced the sumoylation of GST-MEF2C-ΔN2-WT, but not the S396A mutant (Figure 6C). Furthermore, the S396E mutant was more efficiently sumoylated than WT or S396A proteins in the absence of Cdk1/Cyclin B1, and was not further enhanced by Cdk1/Cyclin B1 (Figure 6C). These results suggest that phosphorylation of S396 facilitates the sumoylation of MEF2C-ΔN2 *in vitro*.

We note that the S396A mutation and the K391R/S396A double mutation in the context of the full-length MEF2C appeared to abrogate the transcriptional repression activity of Gal4-MEF2C to similar extents (Fig. 5B). On the other hand, the K391R/S396A double mutation in the context of ΔN2-MEF2C had a significantly larger effect than the S396A mutation in diminishing the transcriptional repression activity of Gal4-ΔN2-MEF2C (Fig. 5C). The underlying reasons for this difference is unclear at present. An intriguing possibility is that, as compared to the full-length MEF2C, sumoylation of ΔN2-MEF2C might be less dependent on prior phosphorylation at S396. Indeed, at later timepoints, ΔN2-MEF2C-S396A was as efficiently sumoylated as ΔN2-MEF2C *in vitro *(Fig. 6B). Unfortunately, the full-length MEF2C did not express well in bacteria, which prohibited us from testing its sumoylation *in vitro*.

We next compared the sumoylation of MEF2C-WT, MEF2C-S396A, and MEF2C-S396E in 10T1/2 cells in the presence of GFP-SUMO1 overexpression (Figure 6D). Consistent with the *in vitro *sumoylation assay, MEF2C-S396A was again less efficiently sumoylated than MEF2C-WT and MEF2C-S396E (Figure 6D). Therefore, these results suggest that sumoylation of MEF2C at K391 is facilitated by phosphorylation of S396 *in vivo*.

## Discussion

Many transcription factors and cofactors can be covalently modified by SUMO [21,22,38]. Sumoylation of these factors generally leads to transcriptional repression. Sumoylation of Elk-1 and p300 facilitates the recruitment of HDAC2, and -6, respectively, suggesting that sumoylation might repress transcription through the recruitment of HDACs to promoters [36,37]. In addition to HDACs, Daxx was shown to be another co-repressor that contributes to SUMO-mediated repression of transcription [44,45].

In this study, we show that MEF2C is sumoylated *in vitro *and *in vivo*, and sumoylation inhibits its transcriptional activity. Sumoylation of MEF2C does not appear to affect its DNA-binding, HDAC-recruitment, nuclear localization [38], stability (data not shown), or p38 phosphorylation, suggesting that sumoylation does not repress the transcriptional activity of MEF2C through these mechanisms. Moreover, when fused to Gal4, MEF2C-ΔN2 that cannot bind to class II HDACs and other transcriptional repressors, including cabin1 [46] and MITR [16], is sufficient to repress the ability of LexA-VP16 to activate transcription from a promoter containing both Gal4- and LexA-binding sites. The K391R mutation abolishes the transcriptional repression activity of Gal4-MEF2C-ΔN2, suggesting that sumoylation of MEF2C-ΔN2 recruits transcriptional repressors other than class II HDACs. Class I HDACs does not seem to be the repressor recruited after sumoylation since overexpression of class I HDACs failed to repress the transcriptional activity of MEF2C in reporter assays (data not shown). Therefore, sumoylation of MEF2C appears to repress its transcriptional activity through a novel mechanism.

### Regulation of the transcriptional activity of MEF2

Transcription activity of myogenic bHLH and MEF2 proteins is tightly regulated during muscle differentiation. Multiple pathways exist to ensure the repression of these transcription factors in dividing myoblasts [47-49]. For example, Cdk4/Cyclin D represses the activity of MEF2 proteins through blocking their interactions with the GRIP1 co-activator [14], although it is unclear whether Cdk4/Cyclin D phosphorylates MEF2 proteins directly. In addition, another cyclin-dependent kinase, Cdk5, phosphorylates MEF2 proteins and inhibits their transcriptional activity in neurons [18].

Consistent with recent findings by Gregoire and Yang [39], we observed that sumoylation-deficient MEF2C has higher myogenic activity than wild-type (Figure 2B and 2C), suggesting that sumoylation might be another important mechanism to actively repress the transcriptional activity of MEF2 proteins in dividing myocytes. Surprisingly, only a very small population of MEF2C is modified by SUMO in C2C12 cells (data not shown). It is unclear how sumoylation of this small population of MEF2C effectively suppresses the activity of cellular MEF2C. On the other hand, this appears to be a recurring theme in the sumoylation of transcriptional factors. One intriguing possibility is that transient sumoylation of these transcriptional factors recruits transcriptional repressors that covalently modify the chromatin at the transcriptional loci, which in turn establishes a relatively long-lived chromatin state that is not permissible for transcription. Alternatively, a "molecular memory" model has been proposed to explain this phenomenon. In this model, a protein molecule that has experienced a sumoylation/desumoylation cycle is proposed to be functionally distinct from one that has never experienced sumoylation [50].

### Regulation of sumoylation of MEF2 by phosphorylation

We have shown that sumoylation of MEF2C appears to be facilitated by phosphorlyation of S396, since S396A mutant is not as efficiently sumoylated *in vivo *and *in vitro *as compared to the wild-type MEF2C. Phosphorylation by Cdk1/Cyclin B1 enhances the sumoylation of MEF2C-ΔN2, but not that of MEF2C-ΔN2-S396A. Surprisingly, though the phospho-mimicking MEF2C-ΔN2-S396E mutant was more efficiently sumoylated than the wild-type *in vitro*, MEF2C-S396E was less efficiently sumoylated than the wild-type *in vivo*. Consistently, MEF2C-S396E was slightly more active than the wild-type in transcriptional assays. The reason for this discrepancy is unclear at present. One possibility is that phosphorylated and sumoylated MEF2C is a better substrate for SUMO isopeptidases *in vivo*. Phosphorylation at S396 of the wild-type MEF2C can be reversed by phosphatases, resulting in longer half-life of sumoylated MEF2C. In contrast, the S396E mutant would be refractory to the actions of phosphatases. Even though MEF2C-S396E can be more efficiently sumoylated as compared to the wild-type, it might be also more prone to desumoylation of SUMO isopeptidases, resulting to its lower steady state of sumoylation *in vivo*.

The kinase that mediates the phosphorylation of S396 *in vivo *is currently unknown. Because S396 is followed by a proline, the S396 kinase is likely to belong to families of proline-directed kinases, such as cyclin-dependent kinases and MAP kinases. Though Cdk1/Cyclin B1 is capable of phosphorylating MEF2C at S396 *in vitro*, we do not think that it is the relevant kinase that phosphorylates MEF2C at this site *in vivo*. Cdk5 was shown to phosphorylate MEF2D at this site in neurons [18]. However, the activity of Cdk5 is strictly dependent on its p35/p39 cofactor that is exclusively expressed in neurons [51]. Furthermore, we did not observe *in vitro *phosphorylation of MEF2C-ΔN2 by Cdk5 alone (data not shown). Thus, yet unidentified kinase(s) mediate phosphorylation of MEF2C at S396 and consequently sumoylation of MEF2C at K391 during muscle differentiation.

There are precedents for phosphorylation-dependent sumoylation. Phosphorylation of HSF1 and PPARγ has recently been shown to facilitate their sumoylation [52,53]. Intriguingly, similar to MEF2C, the phosphorylation sites on HSF1 and PPARγ are both followed by a proline (Figure 6E). In all three cases, the sumoylation and phosphorylation sites are separated by four residues (Figure 6E). Phosphorylation at +5 position by a proline-directed kinase is expected to introduce negative charges at this position, which may enhance the binding affinity between UBC9 and the sumoylation motif. An inspection of the known sumoylation sites reveals that a sumoylation site of c-Myb is also followed by an SP motif at the +5 and +6 positions, suggesting that sumoylation of c-Myb at this site might also be regulated by phosphorylation [31]. In addition, several known sumoylation sites contain an acidic residue (D or E) at the +5 position (Figure 6E). These extended sumoylation consensus motifs that contain acidic residues at +5 position might be more efficiently sumoylated.

## Conclusion

The MEF2 family of transcriptional factors regulates cell proliferation, differentiation, and apoptosis of many cell types. The activities of MEF2 proteins are themselves regulated by multiple mechanisms, including sumoylation. We have discovered that phosphorylation of a serine residue in the vicinity of the sumoylation site facilitates sumoylation of MEF2C. This finding also allows us to define extended sumoylation consensus motifs that contain SP at +5/+6 positions or an acidic residue at +5 position.

## Methods

### Cell culture and transfection

HeLa, 10T1/2, and C2C12 cells were grown in Growth Medium (DMEM supplemented with 10% fetal bovine serum, 100 units/ml penicillin, and 0.1 mg/ml streptomycin). Differentiation of MyoD-transfected 10T1/2 cells was induced by substituting Growth Medium with Differentiation Medium (DMEM supplemented with 2% horse serum, 100 units/ml penicillin, and 0.1 mg/ml streptomycin). Cells were plated and transfected in 12-well plates for promoter-luciferase assays, 6-well plates for immunoprecipitations, 10-cm plates for chromatin immunoprecipitation, and 4-well chambered slides for indirect immunofluorescence microscopy. Cells were transfected using the Effectene (Qiagen) or the Lipofectamine 2000 Plus (Invitrogen) reagents according to manufacturer's protocols.

### Plasmids and promoter assays

The coding regions of human SUMO1 (1–97) and SENP2 were amplified from human fetal thymus cDNA library (BD Biosciences) by PCR and cloned into pCS2 mammalian expression vectors that contain N-terminal Myc, HA, or GFP tags. PIASxβ was amplified from pGEX-PIASxβ constructs provided by S. Muller and cloned into the pCS2-HA vector. UBC9 was cloned into a pCS2 vector that contains a C-terminal Flag tag. The pCDNA-MEF2C, pCDNA-Flag-HDAC4, pCDNA-Flag-HDAC5, and pCDNA-Myc-MyoD constructs were obtained from E. Olson. The pCMV5-HA-p38 and pCMV5-MKK6-DD plasmids were gifts from M. Cobb. The dominant-negative UBC9 mutant and various MEF2C mutants were constructed with the QuikChange site-directed mutagenesis kit (Stratagene). The pET11c-hAOS1, pET28b-hUBA2, and pET28b-hUBC9 vectors were gifts from C. Lima and K. Orth.

Promoter assays were performed in triplicates with the dual-luciferase reporter assay system (Promega) according to manufacturer's protocols. Luciferase activity was measured with a Turner Designs luminometer and normalized for transfection efficiency using the activity of *Renilla *luciferase. The MEF2-responsive promoter activity assays were performed with a pMEF2×3-Luc construct (provided by E. Olson). The Gal4/LexA-promoter activity assay was performed with a pL8G5-Luc plasmid with or without the transfection of a pLexA-VP16 construct.

### Immunofluorescence

10T1/2 fibroblast cells transfected with various plasmids were fixed with 4% paraformaldehyde and permeablized with 0.1% Triton-X100 in PBS. An antibody against myosin heavy chain (MHC) was then added at 1:4 dilutions. After washing, fluorescent secondary antibodies (Molecular Probes) were added at 1:500 dilutions. The cells were washed with PBS, counter-stained with DAPI, and viewed using a 20× objective on a Zeiss Axiovert 200 M microscope. Images were acquired using the Intelligent Imaging software and pseudo-colored in Adobe Photoshop.

### Immunoprecipitation and immunoblotting

Cells were lysed in 400 μl of lysis buffer (50 mM Tris-HCl at pH 7.7, 150 mM KCl, 0.5% NP-40, 5 mM MgCl_2_, 1 mM DTT, 0.5 μM okadaic acid, 10 mM N-ethylmaleimide, supplemented with protease inhibitors; Sigma) for 30 min on ice. After brief sonication, insoluable materials were pelleted by centrifugation at 15,800 × g for 30 min at 4°C. Myc-tagged MEF2C was immunoprecipitated using 0.4 μg of anti-Myc (9E10) monoclonal antibodies (Roche). After incubation at 4°C for 1 hr, 20 μl of Affi-prep protein A beads (Bio-Rad) was added to each lysate and incubated for 1 hr. The beads were washed with lysis buffer and eluted by SDS sample buffer. Eluted proteins were resolved by SDS-PAGE and blotted with anti-Myc (Roche), anti-GFP, or M5-anti-Flag (Sigma) monoclonal antibodies.

### Sumoylation assays

Plasmids that encode appropriate proteins were *in vitro *transcribed and translated (IVT) in reticulocyte lysate in the presence of ^35^S-methionine and subjected to *in vitro *sumoylation reactions, which contained 2 μl of IVT product, 2 μg of AOS1-UBA2, 0.5 μg of UBC9, 1 μg of SUMO1, and 1 μl of Energy Mix (150 mM phosphocreatine, 20 mM ATP, 2 mM EGTA, 20 mM MgCl2, adjust pH to 7.7). Reactions were adjusted to a final volume of 10 μl with the XB buffer (10 mM HEPES, pH 7.7, 1 mM MgCl2, 0.1 mM CaCl2, 100 mM KCl, and 50 mM sucrose). Control reactions contained water and XB buffer. After 2 hr at 30°C, reactions were stopped with 10 μl of 2× SDS sample buffer, boiled, and subjected to SDS-PAGE followed by autoradiography.

### Protein binding, kinase, and chromatin immunoprecipitation assays

For *in vitro *protein binding assays, 4 μg of purified His_6_-p38 was incubated with 5 μl of Ni^2+^-NTA beads in 50 μl of Q-A buffer (20 mM Tris, pH 7.7, 100 mM KCl, 1 mM MgCl_2_) for 1 hr at room temperature. Beads were then incubated with 400 μl of blocking solution (25 mM Tris, pH 8.0, 150 mM NaCl, 2.5 mM KCl, 0.05% Tween-20, 5% dry milk) for 1 hr at RT. 20 μl of *in vitro *sumoylated MEF2C was added and incubated for another 1 hr at RT. Beads were washed two times with blocking solution without dry milk, eluted in 1× SDS sample buffer, and subjected to SDS-PAGE followed by autoradiography.

For p38 *in vitro *kinase assays, 0.4 μg of purified His_6_-p38 was added in 10 μl of 2× Kinase Buffer A (25 mM Tris-HCl, pH 7.7, 100 mM KCl, 5 mM β-glycerophosphate, 10 mM NaF, 1 mM DTT, 0.1 mM Na_3_VO_4_, 10 mM MgCl_2_) containing 200 μM ATP. 10 μl of *in vitro *sumoylated MEF2C was then added and incubated for 30 min at 30°C. The reaction was stopped by the addition of 5 μl of 5× SDS sample buffer and subjected to SDS-PAGE followed by autoradiography. For CDK1 *in vitro *kinase assays, 0.3 μg of purified GST-MEF2C-ΔN2 and 6 μl of purified GST-CDK1/Cyclin B1 (0.2 mg/ml) was incubated for 1 h at 30°C in 10 μl volume containing 1 μl of 10× Kinase Buffer B (25 mM Tris, pH 7.7, 50 mM KCl, 10 mM MgCl_2_, 1 mM DTT), 200 μM ATP, and 0.4 μCi of γ-^32^P-ATP. For sumoylation assays, kinase reactions were performed in the presence of cold ATP. 10 μl of SUMO mix containing 0.1 μg of AOS1-UBA2, 0.05 μg of UBC9, 0.2 μg of His_6_-SUMO1, and 1 μl of Energy Mix in XB buffer was added to the kinase reaction mixture. The reaction was further incubated for 15 min at 30°C and stopped by the addition of 20 μl of 2× SDS sample buffer.

Chromatin immunoprecipitation (ChIP) assays were performed with a ChIP assay kit (Upstate) according to the manufacturer's protocol.

## List of abbreviations

MEF2, myocyte enhancer factor 2; HDAC. histone deacetylase; SUMO, small ubiquitin-like modifier; ChIP, chromatin immunoprecipitation; GFP, green fluorescent protein.

## Authors' contributions

JK designed and performed most of the experiments and wrote the paper. CBG contributed key reagents and participated in experimental design. HY contributed to data interpretation and manuscript preparation.

## Supplementary Material

Additional File 1 **Sumoylation of MEF2C does not affect its DNA-binding, HDAC-interaction, or p38 phosphorylation**. **(A) **MEF2C-WT- or MEF2C-K391R-expressing plasmids were co-transfected with a *MEF2*×3-luciferase reporter construct into HeLa cells. The total lystates were immunoprecipitated with anti-GFP (negative control) or anti-MEF2C beads. DNA in total lysates or bound to beads was subjected to PCR with primers that annealed to the promoter region of the reporter gene and analyzed by electrophoresis in 1% agarose gel. **(B) **HeLa cells were transfected with the indicated plasmids. The total cell lysates and the anti-Myc immunoprecipitates were resolved by SDS-PAGE and blotted with anti-Myc or anti-Flag. **(C) ***In vitro *translated ^35^S-labeled MEF2C was incubated with or without SUMO reaction mixtures and then mixed with Ni^2+^-NTA beads containing His_6_-p38. The proteins bound to beads were resolved by SDS-PAGE followed by autoradiography. **(D) ***In vitro *translated MEF2C was incubated with or without SUMO reaction mixtures and then subjected to p38 kinase assays. The samples were resolved by SDS-PAGE followed by autoradiography. **(E) ***In vitro *translated Myc-MEF2C was incubated with SUMO reaction mixtures and then subjected to p38 kinase assays. Myc-MEF2C was immunoprecipitated with anti-Myc and incubated with Lambda phosphatase. The samples were resolved by SDS-PAGE followed by autoradiography. **(F) **HeLa cells were transfected with the indicated plasmids. The total cell lysates were resolved by SDS-PAGE and blotted with anti-Myc or anti-HA. Click here for file

## References

[B1] McKinsey TA, Zhang CL, Olson EN (2002). MEF2: a calcium-dependent regulator of cell division, differentiation and death. Trends Biochem Sci.

[B2] Black BL, Olson EN (1998). Transcriptional control of muscle development by myocyte enhancer factor-2 (MEF2) proteins. Annu Rev Cell Dev Biol.

[B3] Han A, He J, Wu Y, Liu JO, Chen L (2005). Mechanism of recruitment of class II histone deacetylases by myocyte enhancer factor-2. J Mol Biol.

[B4] Han A, Pan F, Stroud JC, Youn HD, Liu JO, Chen L (2003). Sequence-specific recruitment of transcriptional co-repressor Cabin1 by myocyte enhancer factor-2. Nature.

[B5] Lin Q, Schwarz J, Bucana C, Olson EN (1997). Control of mouse cardiac morphogenesis and myogenesis by transcription factor MEF2C. Science.

[B6] Lin Q, Lu J, Yanagisawa H, Webb R, Lyons GE, Richardson JA, Olson EN (1998). Requirement of the MADS-box transcription factor MEF2C for vascular development. Development.

[B7] Naya FJ, Black BL, Wu H, Bassel-Duby R, Richardson JA, Hill JA, Olson EN (2002). Mitochondrial deficiency and cardiac sudden death in mice lacking the MEF2A transcription factor. Nat Med.

[B8] Wang L, Fan C, Topol SE, Topol EJ, Wang Q (2003). Mutation of MEF2A in an inherited disorder with features of coronary artery disease. Science.

[B9] Lee Y, Nadal-Ginard B, Mahdavi V, Izumo S (1997). Myocyte-specific enhancer factor 2 and thyroid hormone receptor associate and synergistically activate the alpha-cardiac myosin heavy-chain gene. Mol Cell Biol.

[B10] Molkentin JD, Black BL, Martin JF, Olson EN (1995). Cooperative activation of muscle gene expression by MEF2 and myogenic bHLH proteins. Cell.

[B11] Blaeser F, Ho N, Prywes R, Chatila TA (2000). Ca(2+)-dependent gene expression mediated by MEF2 transcription factors. J Biol Chem.

[B12] Morin S, Charron F, Robitaille L, Nemer M (2000). GATA-dependent recruitment of MEF2 proteins to target promoters. EMBO J.

[B13] Sartorelli V, Huang J, Hamamori Y, Kedes L (1997). Molecular mechanisms of myogenic coactivation by p300: direct interaction with the activation domain of MyoD and with the MADS box of MEF2C. Mol Cell Biol.

[B14] Lazaro JB, Bailey PJ, Lassar AB (2002). Cyclin D-cdk4 activity modulates the subnuclear localization and interaction of MEF2 with SRC-family coactivators during skeletal muscle differentiation. Genes Dev.

[B15] Miska EA, Karlsson C, Langley E, Nielsen SJ, Pines J, Kouzarides T (1999). HDAC4 deacetylase associates with and represses the MEF2 transcription factor. EMBO J.

[B16] Youn HD, Grozinger CM, Liu JO (2000). Calcium regulates transcriptional repression of myocyte enhancer factor 2 by histone deacetylase 4. J Biol Chem.

[B17] Kato Y, Zhao M, Morikawa A, Sugiyama T, Chakravortty D, Koide N, Yoshida T, Tapping RI, Yang Y, Yokochi T, Lee JD (2000). Big mitogen-activated kinase regulates multiple members of the MEF2 protein family. J Biol Chem.

[B18] Gong X, Tang X, Wiedmann M, Wang X, Peng J, Zheng D, Blair LA, Marshall J, Mao Z (2003). Cdk5-mediated inhibition of the protective effects of transcription factor MEF2 in neurotoxicity-induced apoptosis. Neuron.

[B19] Zhao M, New L, Kravchenko VV, Kato Y, Gram H, di Padova F, Olson EN, Ulevitch RJ, Han J (1999). Regulation of the MEF2 family of transcription factors by p38. Mol Cell Biol.

[B20] Zhu B, Gulick T (2004). Phosphorylation and alternative pre-mRNA splicing converge to regulate myocyte enhancer factor 2C activity. Mol Cell Biol.

[B21] Johnson ES (2004). Protein modification by SUMO. Annu Rev Biochem.

[B22] Gill G (2004). SUMO and ubiquitin in the nucleus: different functions, similar mechanisms?. Genes Dev.

[B23] Sachdev S, Bruhn L, Sieber H, Pichler A, Melchior F, Grosschedl R (2001). PIASy, a nuclear matrix-associated SUMO E3 ligase, represses LEF1 activity by sequestration into nuclear bodies. Genes Dev.

[B24] Kotaja N, Karvonen U, Janne OA, Palvimo JJ (2002). PIAS proteins modulate transcription factors by functioning as SUMO-1 ligases. Mol Cell Biol.

[B25] Pichler A, Gast A, Seeler JS, Dejean A, Melchior F (2002). The nucleoporin RanBP2 has SUMO1 E3 ligase activity. Cell.

[B26] Kagey MH, Melhuish TA, Wotton D (2003). The polycomb protein Pc2 is a SUMO E3. Cell.

[B27] Ross S, Best JL, Zon LI, Gill G (2002). SUMO-1 modification represses Sp3 transcriptional activation and modulates its subnuclear localization. Mol Cell.

[B28] Sapetschnig A, Rischitor G, Braun H, Doll A, Schergaut M, Melchior F, Suske G (2002). Transcription factor Sp3 is silenced through SUMO modification by PIAS1. EMBO J.

[B29] Poukka H, Karvonen U, Janne OA, Palvimo JJ (2000). Covalent modification of the androgen receptor by small ubiquitin-like modifier 1 (SUMO-1). Proc Natl Acad Sci U S A.

[B30] Abdel-Hafiz H, Takimoto GS, Tung L, Horwitz KB (2002). The inhibitory function in human progesterone receptor N termini binds SUMO-1 protein to regulate autoinhibition and transrepression. J Biol Chem.

[B31] Bies J, Markus J, Wolff L (2002). Covalent attachment of the SUMO-1 protein to the negative regulatory domain of the c-Myb transcription factor modifies its stability and transactivation capacity. J Biol Chem.

[B32] Kim J, Cantwell CA, Johnson PF, Pfarr CM, Williams SC (2002). Transcriptional activity of CCAAT/enhancer-binding proteins is controlled by a conserved inhibitory domain that is a target for sumoylation. J Biol Chem.

[B33] Tian S, Poukka H, Palvimo JJ, Janne OA (2002). Small ubiquitin-related modifier-1 (SUMO-1) modification of the glucocorticoid receptor. Biochem J.

[B34] Subramanian L, Benson MD, Iniguez-Lluhi JA (2003). A synergy control motif within the attenuator domain of CCAAT/enhancer-binding protein alpha inhibits transcriptional synergy through its PIASy-enhanced modification by SUMO-1 or SUMO-3. J Biol Chem.

[B35] Yang SH, Jaffray E, Hay RT, Sharrocks AD (2003). Dynamic interplay of the SUMO and ERK pathways in regulating Elk-1 transcriptional activity. Mol Cell.

[B36] Yang SH, Sharrocks AD (2004). SUMO promotes HDAC-mediated transcriptional repression. Mol Cell.

[B37] Girdwood D, Bumpass D, Vaughan OA, Thain A, Anderson LA, Snowden AW, Garcia-Wilson E, Perkins ND, Hay RT (2003). P300 transcriptional repression is mediated by SUMO modification. Mol Cell.

[B38] Gocke C, Yu H, Kang J (2004). Systematic identification and analysis of mammalian small ubiquitin-like modifier substrates. J Biol Chem.

[B39] Gregoire S, Yang XJ (2005). Association with class IIa histone deacetylases upregulates the sumoylation of MEF2 transcription factors. Mol Cell Biol.

[B40] Zhao X, Sternsdorf T, Bolger TA, Evans RM, Yao TP (2005). Regulation of MEF2 by histone deacetylase 4- and SIRT1 deacetylase-mediated lysine modifications. Mol Cell Biol.

[B41] Rodriguez MS, Dargemont C, Hay RT (2001). SUMO-1 conjugation in vivo requires both a consensus modification motif and nuclear targeting. J Biol Chem.

[B42] Bernier-Villamor V, Sampson DA, Matunis MJ, Lima CD (2002). Structural basis for E2-mediated SUMO conjugation revealed by a complex between ubiquitin-conjugating enzyme Ubc9 and RanGAP1. Cell.

[B43] McKinsey TA, Zhang CL, Olson EN (2001). Control of muscle development by dueling HATs and HDACs. Curr Opin Genet Dev.

[B44] Lin DY, Fang HI, Ma AH, Huang YS, Pu YS, Jenster G, Kung HJ, Shih HM (2004). Negative modulation of androgen receptor transcriptional activity by Daxx. Mol Cell Biol.

[B45] Chang CC, Lin DY, Fang HI, Chen RH, Shih HM (2005). Daxx mediates the small ubiquitin-like modifier-dependent transcriptional repression of Smad4. J Biol Chem.

[B46] Youn HD, Sun L, Prywes R, Liu JO (1999). Apoptosis of T cells mediated by Ca2+-induced release of the transcription factor MEF2. Science.

[B47] Tapscott SJ, Davis RL, Thayer MJ, Cheng PF, Weintraub H, Lassar AB (1988). MyoD1: a nuclear phosphoprotein requiring a Myc homology region to convert fibroblasts to myoblasts. Science.

[B48] Montarras D, Chelly J, Bober E, Arnold H, Ott MO, Gros F, Pinset C (1991). Developmental patterns in the expression of Myf5, MyoD, myogenin, and MRF4 during myogenesis. New Biol.

[B49] Breitbart RE, Liang CS, Smoot LB, Laheru DA, Mahdavi V, Nadal-Ginard B (1993). A fourth human MEF2 transcription factor, hMEF2D, is an early marker of the myogenic lineage. Development.

[B50] Hay RT (2005). SUMO: a history of modification. Mol Cell.

[B51] Dhavan R, Tsai LH (2001). A decade of CDK5. Nat Rev Mol Cell Biol.

[B52] Yamashita D, Yamaguchi T, Shimizu M, Nakata N, Hirose F, Osumi T (2004). The transactivating function of peroxisome proliferator-activated receptor gamma is negatively regulated by SUMO conjugation in the amino-terminal domain. Genes Cells.

[B53] Hietakangas V, Ahlskog JK, Jakobsson AM, Hellesuo M, Sahlberg NM, Holmberg CI, Mikhailov A, Palvimo JJ, Pirkkala L, Sistonen L (2003). Phosphorylation of serine 303 is a prerequisite for the stress-inducible SUMO modification of heat shock factor 1. Mol Cell Biol.

