# Supplementary figures and images for: Phosphorylation-facilitated sumoylation of MEF2C negatively regulates its transcriptional activity
Source: BMC Biochem. 2006 Feb 14;7:5. doi: 10.1186/1471-2091-7-5 (PMC1386686; doi:10.1186/1471-2091-7-5)

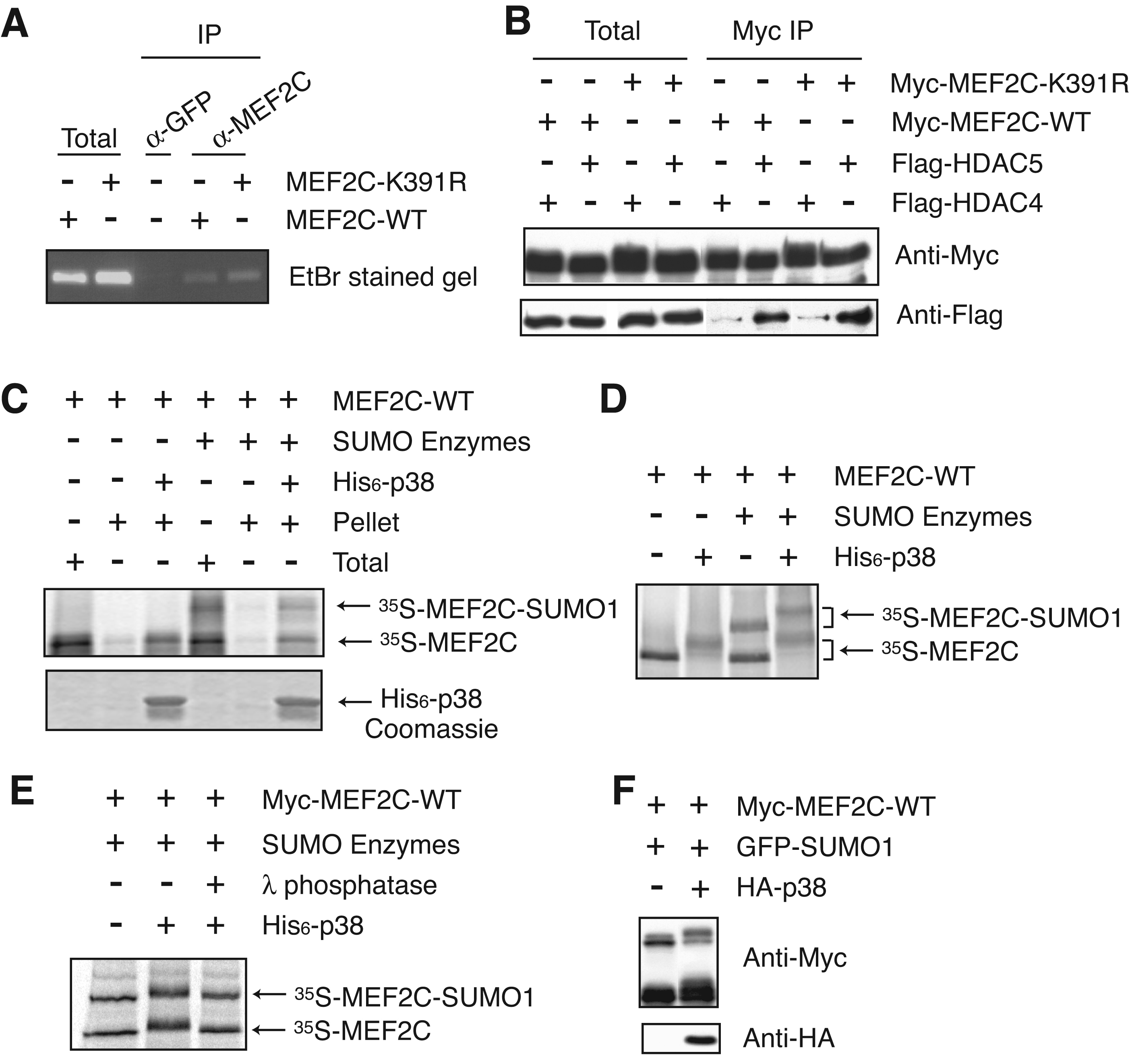

Supplement: Additional File 1 — Sumoylation of MEF2C does not affect its DNA-binding, HDAC-interaction, or p38 phosphorylation. (A) MEF2C-WT- or MEF2C-K391R-expressing plasmids were co-transfected with a MEF2×3-luciferase reporter construct into HeLa cells. The total lystates were immunoprecipitated with anti-GFP (negative control) or anti-MEF2C beads. DNA in total lysates or bound to beads was subjected to PCR with primers that annealed to the promoter region of the reporter gene and analyzed by electrophoresis in 1% agarose gel. (B) HeLa cells were transfected with the indicated plasmids. The total cell lysates and the anti-Myc immunoprecipitates were resolved by SDS-PAGE and blotted with anti-Myc or anti-Flag. (C) In vitro translated 35S-labeled MEF2C was incubated with or without SUMO reaction mixtures and then mixed with Ni2+-NTA beads containing His6-p38. The proteins bound to beads were resolved by SDS-PAGE followed by autoradiography. (D) In vitro translated MEF2C was incubated with or without SUMO reaction mixtures and then subjected to p38 kinase assays. The samples were resolved by SDS-PAGE followed by autoradiography. (E) In vitro translated Myc-MEF2C was incubated with SUMO reaction mixtures and then subjected to p38 kinase assays. Myc-MEF2C was immunoprecipitated with anti-Myc and incubated with Lambda phosphatase. The samples were resolved by SDS-PAGE followed by autoradiography. (F) HeLa cells were transfected with the indicated plasmids. The total cell lysates were resolved by SDS-PAGE and blotted with anti-Myc or anti-HA. [file 1471-2091-7-5-S1.jpeg]
